# The Use of Tuning Forks for Studying Behavioural Responses in Orb Web Spiders

**DOI:** 10.3390/insects13040370

**Published:** 2022-04-09

**Authors:** Mollie S. Davies, Thomas Hesselberg

**Affiliations:** 1 Department of Biological and Medical Sciences, Headington Campus, Oxford Brookes University, Oxford OX3 0BP, UK; mdavies08@qub.ac.uk; 2 Department of Zoology, University of Oxford, 11 Mansfield Road, Oxford OX1 3SZ, UK

**Keywords:** prey capture behaviour, anti-predatory behaviour, tetragnatha, ethogram, vibration

## Abstract

**Simple Summary:**

Spiders are common predators found in almost every type of environment, and are used as model organisms in studies ranging from communication and signalling to biochemical studies on their silk. Orb spiders are particularly interesting, as their web provides a cost-effective way to obtain information on their foraging behaviour. However, studies on short-term behaviours including prey capture and escape behaviours are rare and usually take place in artificial settings, such as laboratories. In this study, we tested a simple methodology using tuning forks that can be used consistently and reliably in the field. The two tuning forks are capable of producing attack (440 Hz) and escape (256 Hz) responses from the spiders. We also used a metal wire as a mechanical stimulus for comparison, which as predicted, was less reliable. We demonstrate the usefulness of the methodology by quantitatively investigating how the size of the spider and the size of its web affect predatory and escape response rates in the autumn spider, although no significant effects of either were found. However, our results confirm the ease by which this simple method can be used to conduct behavioural studies of orb spiders in the wild.

**Abstract:**

Spiders and their webs are often used as model organisms to study a wide range of behaviours. However, these behavioural studies are often carried out in the laboratory, and the few field studies usually result in large amounts of video footage and subsequent labour-intensive data analysis. Thus, we aimed to devise a cost- and time-effective method for studying the behaviour of spiders in the field, using the now almost forgotten method of stimulating webs with tuning forks. Our study looked at the viability of using 256 Hz and 440 Hz tuning forks to stimulate, anti-predatory and predatory responses in the orb web spider *Metellina segmentata*, respectively. To assess the consistency of the behaviours produced, we compared these to direct mechanical stimulation with a metal wire. The results suggest that the tuning forks produce relatively consistent behaviours within and between two years in contrast to the metal wire. We furthermore found no significant effects of spider length or web area on spider reaction times. However, we found significant differences in reaction times between escape and prey capture behaviours, and between tuning forks and the wire. Thus, we demonstrated the potential of tuning forks to rapidly generate quantitative data in a field setting.

## 1. Introduction

Spiders are the most well-known, most diverse, and arguably the most interesting of the Arachnida. This has led to their use as model organisms in a wide variety of studies, ranging from animal signalling and communication [1] and invertebrate cognition [2] to biomechanical studies on silk [3]. Those that build webs are particularly interesting as, by using their webs as extended phenotypes, they are capable of detecting and responding to a wide variety of stimuli [4,5]. Orb spiders are the best-studied web-building spiders for a number of reasons; (i) they are abundant and widespread in most ecosystems, (ii) their highly structured two-dimensional webs are easy to quantify in both the laboratory and in the field [6,7], and (iii) their webs show impressive plasticity in their geometrical structure in response to a wide range of physiological and environmental factors [8,9].

Further, the use of spiders in behavioural studies is supported by sufficient documentation of their anti-predatory and predatory behaviours, from families to specific species. The anti-predatory responses of spiders are best summarised by Cloudsley-Thompson [10]. Their predators fall into two main categories, similar-sized arthropods, and vertebrates that are much larger. They employ a range of primary and secondary defences against each predator type [10]. Primary defences reduce the chances of a predator encounter and include living in crevices, beneath bark or within holes (anachoresis), crypsis/protective colouration, and phenological adaptations [10]. Secondary defences are more active, and in orb web spiders, they commonly include flight, thanatosis and rebuff. Flight involves the spider dropping from the web, which becomes more successful when followed by thanatosis and/or crypsis. The behaviour of rebuff was first described by Tolbert [11], during an experiment conducted on *Argiope aurantia* (Lucas, 1833) and *Argiope trifasciata* (Forsskål, 1775), and involves the spiders actively pushing away the stimulus using their front legs. When acting as a predator, spiders rely on tactile and vibratory cues, detected through the surface of a leaf or web silk [12]. The predatory tactics of spiders include active pursuit, sit-and-wait, prey attraction and cautious stalking. However, their dependence on vibratory cues to reveal prey means sit-and-wait predatory approaches are the most common [12]. Orb spiders’ prey-capture strategy consists of an initial reaction of turning towards prey caught in the web followed by gathering information on the location and possible size of the prey based on vibrations generated by the struggling prey, which is occasionally supplemented by plucking the threads with its first pair of legs [13,14]. The spider then approaches the prey before attacking it by either biting and/or wrapping it with silk [14]. However, these behaviours are almost exclusively studied under unnatural conditions in the laboratory (examples include [15,16,17]). Field studies on these naturally rare events remain scarce, and when they do occur, the methods usually involve large quantities of video footage, and subsequently, time-consuming analysis [18,19] (but see [20]). Thus, to obtain an integrated view of spider behaviour in the wild, there is a need to develop an easier and more reliable method. Here, we suggest that one such cost-effective method could be the now almost forgotten practice of stimulating behavioural responses with tuning forks contacting the web thread.

This method takes advantage of the highly geometrical structure of the standard orb web with radii radiating outwards from the hub towards the peripheral frame threads, which enclose the web with sticky spiral threads overlain on the radii [21]. The stronger and stiffer radii and frame threads transmit vibratory information from struggling prey to the spider waiting in the hub of the web, and are therefore the ideal threads to stimulate with tuning forks [22,23]. The use of tuning forks to study spiders has a long history, with Boys’ study from 1880 [24] being one of the earliest documented examples. Boys approached orb web garden spiders (species not given, but likely *Araneus diadematus* (Clerck, 1757) in the family Araneidae) with a 440 Hz tuning fork and observed the spiders facing and approaching the tuning fork—a clear demonstration of predatory behaviour [24]. In a more elaborate experiment, Barrows [25] studied the effect of three tuning forks (100 Hz, 487 Hz and an adjustable fork) and an electric vibrator on the orb weaving spider *Larinioides sclopetarius* (Clerck, 1757) in the family Araneidae. It was concluded that the spider orientated itself and moved toward the stimulus when it was vibrating at an appropriate rate and amplitude [25]. It was also determined that the sensory organs used in detecting the vibrating stimulus are likely to be mechanosensitive trichobothria found on the tarsus and metatarsus of the legs [25,26]. Bays [27] conditioned the orb web spider *Araneus diadematus* (Clerck, 1757) in the family Araneidae to respond to different prey options, which were associated with two frequencies, 256 Hz and 523.3 Hz. His experiment revealed that orb web spiders are able to distinguish between different vibrational frequencies, and that they adapt their behaviour based on previous experience [27]. More recently, Justice et al. [28] studied the orb web spider *Argiope florida* (Chamberlin and Ivie, 1944) in the family Araneidae and elicited predatory behaviours using a frequency of 100 Hz. Whilst in an experiment conducted by Nakata and Mori [29], a 440 Hz frequency was used to produce anti-predatory web-building behaviours (webs were more symmetrical in the presence of predator cues) in *Cyclosa argenteoalba* (Bösenberg and Strand, 1906) and *Eriophora sagana* (Bösenberg and Strand, 1906), both in the family Araneidae.

This historic use of tuning forks demonstrates that they are useful for studying the predatory and anti-predatory behaviours of orb web spiders. However, the majority of the studies mentioned above were conducted in a laboratory setting, and only medium-to-large-sized araneid orb spiders were used. This, combined with the variability in the behavioural responses generated by different species and different tuning forks, suggests there is a need for more rigorous and quantitative studies on a wide range of species. Therefore, in this study, we investigated whether two different tuning forks (256 Hz and 440 Hz) could be used to produce anti-predatory and prey-capture behavioural responses in the orb web spider *Metellina segmentata* (Clerck, 1757) from the family Tetragnathidae, in the field. Additionally, we compared the consistency of the behavioural responses to tuning forks to those from a mechanical stimulation of the spider with a wire. The practical use of the tuning forks was also demonstrated to generate quantitative data by comparing the spider’s behavioural response against both the size of the spider and the web. 

## 2. Materials and Methods

### 2.1. Study Sites

The following localities situated in Southeast Wales were visited for data collection: Allt-yr-yn (51°35′37″ N, 003°00′48″ W. 74 m above sea level), Bargoed Country Park (51°41′00.1″ N 3°13′30.4″ W, 164 m a.s.l.), Cwmcarn Forest (51°38′13.1″ N 3°05′53.3″ W, 193 m a.s.l.), Magor Marsh Reserve (51°34′31.2″ N 2°49′41.7″ W, 5 m a.s.l.) and Wentwood (51°38′31.2″ N 2°48′38.4″ W, 203 m a.s.l.). These sites were chosen based on their accessibility and historic records which indicated the presence of *M. segmentata*. The climatic variables at our studies sites during our survey days (August–September in both years) ranged from a minimum average temperature of 16.9 °C (Allt-yr-yn) to a maximum of 25.9 °C (Magor Marsh) in 2020 and from a minimum average temperature of 17.9 °C (Allt-yr-yn) to a maximum of 21.8 °C (Magor Marsh) in 2021, while the average relative humidity ranged from a minimum of 73.6% (Bargoed Country Park) to a maximum of 80.6% (Allt-yr-yn) in 2020 and a minimum of 71.0% (Cwmcarn Forest) to a maximum of 78.5% (Allt-yr-yn) in 2021.

### 2.2. Study Species

*Metellina segmentata* is a small-to-medium-sized orb web spider (adult females are 4–9 mm long) in the Tetragnathidae family [30], which, similarly to its sister species *Metellina mengei* (Blackwall, 1870), construct horizontally inclined orb webs, and is often found waiting for prey in the centre of its hub during the day [31]. *M. segmentata* and *M. mengei* are difficult to differentiate in the field, but as the latter is only active in the spring, we assumed all the adult females tested in this study were *M. segmentata*. A few individuals from each location were also taken to back to the laboratory and confirmed as *M. segmentata*.

### 2.3. Data Collection

We used two tuning forks (256 Hz and 440 Hz) and an 8 cm length of wire to study the behavioural responses of *M. segmentata*. The frequencies of the tuning forks were chosen to be within the range of previously studied tuning forks, with the expected responses based on observations that smaller prey insects, such as mosquitoes, have higher wingbeat frequencies (500–650 Hz) [32], while larger, and potentially more dangerous insects, such as bees and wasps, have lower wing beat frequencies (100–250 Hz) [33]. We included the wire as we expected the direct physical stimulation to act as a simulation of larger vertebrate predators, and hence potentially to produce a stronger and more consistent anti-predator response. Therefore, our expectations were that 440 Hz would tend to generate predatory responses, while 256 Hz and the wire would generate anti-predatory behaviours.

Data were obtained over two years (2020 and 2021) using slightly different methods. In 2020, data collection took place between the 8th of August and the 6th of September and consisted of using the stimuli in the same order every time; first the 256 Hz tuning fork, followed by the 440 Hz tuning fork, and finally the wire. In 2021, data were collected between the 1st of August and the 17th of August and here we randomized the order in which the stimuli were presented to each spider by using a random number generator prior to going out into the field.

The processes of both methods were the same, with the order in which stimuli were presented to the individual spider being the only difference. After locating the spider in the hub of its web, the tuning forks would be hit on an object (i.e., a plastic spray water bottle) to generate vibrations, and the fork would then be brought slowly towards the web to touch its frame. The wire would touch the spiders directly on the back of its abdomen. Between each stimulus, a minimum waiting period of 5 min and a maximum wait time of 20 min was allowed for the spiders to return to the hub if they had responded by escaping or attacking. Most spiders returned within this time frame. Our methodology was similar to that of Boys [24], Bays [27] and Justice et al. [28].

To quantify the behavioural responses, the entire response of the spider was written down in the field, and used to create an ethogram (Table 1). This ethogram consisted of three broad categories of behaviour, (i) attack, (ii) escape and (iii) no response—these terms will subsequently be used throughout this report.

The spiders were filmed using a Canon EOS 1300D camera (Canon Inc., Ōta, Tokyo Metropolis, Japan) and a Canon EF-S 18–55 mm Macro Lens (Canon Inc., Ōta, Tokyo Metropolis, Japan) —0.25 m/0.8 ft—with a frame rate of 25 fps. Videos S1–S4 recordings allowed us to clarify behavioural responses and enabled us to measure the spiders’ reaction to the stimulation. The reaction time was measured from when the stimulus came into contact with the web or spider, to the initiation of the spider’s behavioural response. For example, if the spider turned then moved towards the direction of the tuning fork, the reaction time was measured from when the tuning fork came into contact with the web, to when the spider first turned. If the spider dropped from the web before the tuning fork made contact, this resulted in a negative reaction time.

As well as behavioural responses and reaction times, the length of the spider (mm), height (cm) and length (cm) of the capture spiral were measured using a standard 30 cm ruler. The capture spiral area was calculated using the ellipse-hub equation (vertical diameter/2 × horizontal diameter/2 × π—(hub diameter/2)2) [34]. The temperature (°C) and humidity (%rH) were measured using an ETI 810-190 Pocket-Sized Thermo Hygrometer (Electronical Temperature Instruments Ltd., Worthing, U.K.)).

### 2.4. Data Analysis

For the 2020 method, a total of 50 spiders were observed during the initial data collection, and 38 were subsequently used in the data analysis. For the 2021 method, 46 spiders were observed during the initial data collection, and 40 were used in the subsequent data analysis. Spiders were removed due to insufficient data, primarily caused by not returning between stimuli. A Fisher’s Exact test for contingency tables [35] was used to compare frequency distribution across the different stimuli and across the years, as well as between the order in which the stimuli were presented, as a Chi-square contingency test could not be used due to many expected values being below 5. We developed linear mixed models (using the *lmer()* function from the lme4 package) in R [36] to test whether the type of behavioural response (using only the data where the spider responded either by attacking or escaping) varied with spider size and capture spiral area, where the latter were response variables and behavioural response a fixed variable. Site and year were included as random variables. 

For all models, we log-transformed the response variables to achieve normality of the residuals and *p* values were estimated from the Type II Wald F tests with Kenward–Roger degrees of freedom. We used R [37] for all statistical tests with a significance level of 0.05.

## 3. Results

### 3.1. Behavioural Responses

The produced ethogram shows that our spiders displayed a wide range of responses to the different stimuli, which we could categorise into either attack, escape, or no response behaviours (Table 1). The attack behaviours were the most complex of the three categories and generally involved moving towards the stimuli and in some instances physically interacting with the tuning fork or wire. Interestingly, unlike for escape or no response behaviours, the spiders reacted to the wire with different attack behaviours than they did to the tuning forks. The most common reaction to the tuning fork was an initial aggressive movement towards the tuning fork, but either pausing or dropping from the web before it came into contact with it. The most frequent escape response was to rapidly drop off the web (Table 1). The small proportion of spiders that did not respond mainly showed no visible reaction, although a few flinched slightly (Table 1). 

**Figure 1:**
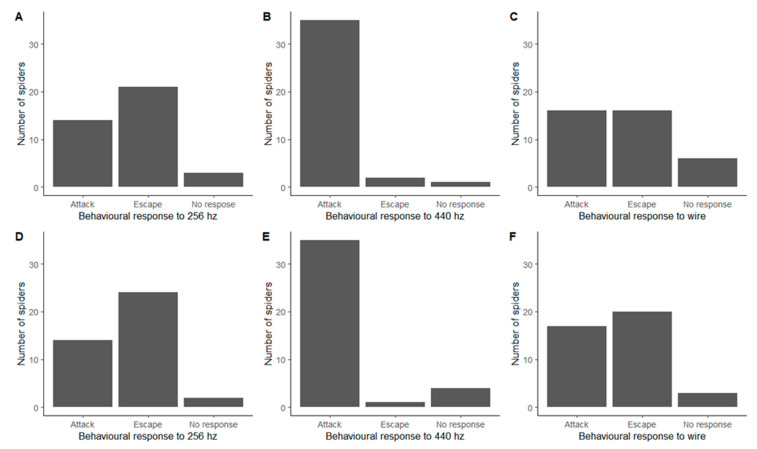
Behavioural responses of *Metellina segmentata*, showing the number of attack, escape, and no response behaviours seen for each stimulus. Split into 2020 method (**A**–**C**) and 2021 method (**D**–**F**).

In order to better quantify spiders’ reactions, we analysed the behaviours in the larger categories, attack, escape, or no response. We found a significant difference in the responses to the three stimuli for both 2020 (Fisher’s Exact Test, *p* < 0.0001) and 2021 (Fisher’s Exact Test, *p* < 0.0001), with spiders predominantly escaping in response to the 256 Hz tuning fork, almost exclusively attacking in response to the 440 Hz and either attacking or escaping in response to the wire (Figure 1). The number of spiders not responding remained low for all stimuli, with the highest proportion of 16% found for the wire in 2020 (Table 1).

The behavioural responses from the 2020 method and 2021 method were consistent with each other (Figure 1). There were no significant differences in the frequency distributions of responses between 2020 and 2021 for the 256 Hz tuning fork (Fisher’s Exact Test, *p* = 0.81), the 440 Hz tuning fork (Fisher’s Exact Test, *p* = 0.51) or the wire (Fisher’s Exact Test, *p* = 0.49).

### 3.2. Order of Stimuli

**Figure 2:**
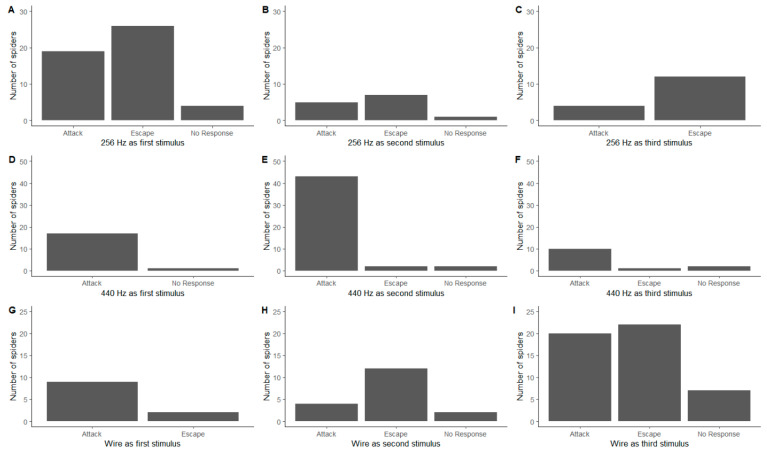
The influence of the order of the stimuli on the behavioural responses of *Metellina segmentata*. (**A**–**C**) 256 Hz tuning fork; (**D**–**F**) 440 Hz tuning fork; (**G**–**I**) length of wire.

We found no clear influence of the order of stimuli (which was consistent in 2020 and randomised in 2021), as behavioural responses were similar independently of the order in which they were presented to the spider for both the 256 Hz (Figure 2A–C; Fisher’s Exact Test, *p* = 0.584) and the 440 Hz tuning fork (Figure 2D–F; Fisher’s Exact Test, *p* = 0.333). However, the behavioural responses to the wire showed less consistency (Figure 2G–I; Fisher’s Exact Test, *p* = 0.033). When the wire used as the first stimulus, the primary response was to attack (Figure 2G), whilst when it was the second stimulus, the primary response was to escape (Figure 2H). These results are further confounded by almost equal attack and escape behavioural responses when it was the third stimulus (Figure 2I).

### 3.3. Effect of Size and Web Area

**Figure 3:**
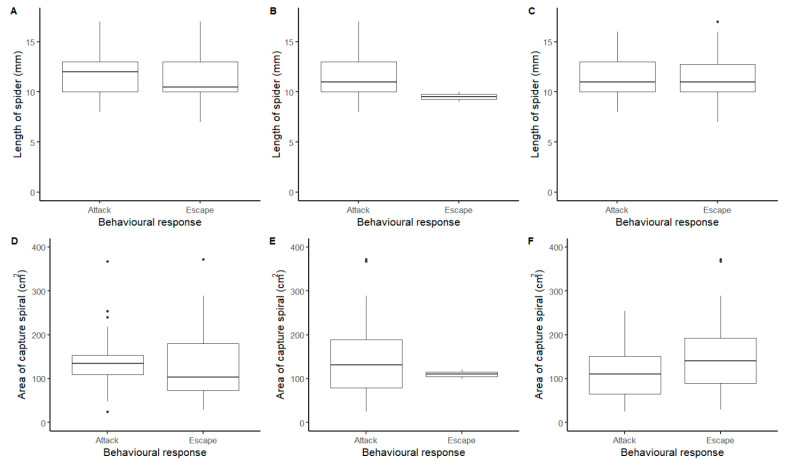
Comparison of the length of the spider (mm) and the area of the capture spiral (cm^2^) against the behavioural responses observed in *Metellina segmentata.* (**A**,**D**) 256 Hz tuning fork; (**B**,**E**) 440 Hz tuning fork; (**C**,**F**) length of wire.

In order to showcase some of the quantitative questions that can be answered using these methods, we compared behavioural responses (attack or escape only) between spiders of different sizes and with different sized webs for the full data-set, disregarding year and order of stimuli as they had no large impact on the behavioural response as demonstrated above. We found that statistically, the length of the spider did not differ between behavioural responses to 256 Hz (F = 2.41, df = 1, 67.5, *p* = 0.13), 440 Hz (F = 1.20, df = 1, 68.5, *p* = 0.28), or the wire (LMM: F = 0.003, df = 1, 63.8, *p* = 0.96). Similar results were obtained with web area, where we also found no differences in behavioural response to 256 Hz (F = 1.22, df = 1, 67.8, *p* = 0.27), 440 Hz (F = 0.47, df = 1, 69.1, *p* = 0.49) or the wire (F = 2.57, df = 1, 63.4, *p* = 0.11). Nonetheless, the average length of spiders and the average capture spiral area tended both to be larger in spiders that attacked (Figure 3). Whilst for the wire, the size of the spider had no impact on the behavioural response observed, although spiders that escaped tended to have a larger capture spiral (Figure 3).

### 3.4. Reaction Times

**Figure 4:**
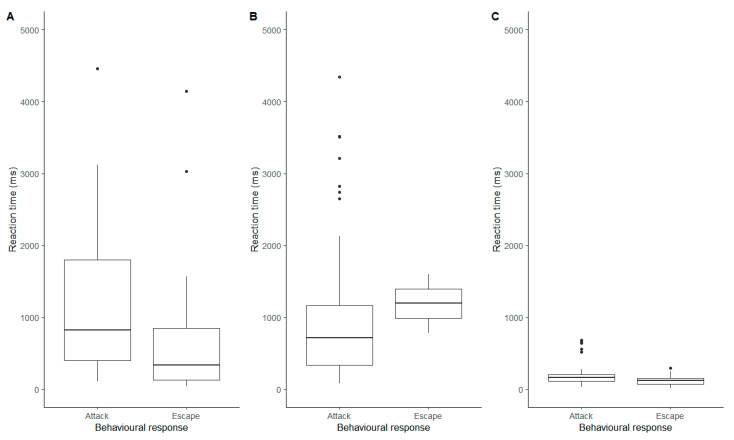
Reaction times (ms) of the attack and escape behavioural responses of *Metellina segmentata* for the three stimuli. (**A**) 256 Hz tuning fork; (**B**) 440 Hz tuning fork; (**C**) Metal wire.

The stimulus type (tuning forks or wire) and the behavioural response (attack or escape) both had a significant impact on the reaction time of the spider (Behaviour: F = 22.8, df = 1, 202.0, *p* < 0.0001; Stimulus: F = 4.09, df = 2, 200.3, *p* = 0.018) and the interaction between them was also significant (F = 9.47, df = 2, 201.5, *p* = 0.0001). Interestingly, whilst spiders had faster reactions when escaping the 256 Hz tuning fork, they reacted faster when they attacked the 440 Hz tuning (Figure 4). In response to the wire, the difference was minimal, although there was a slight tendency for them to react faster when escaping (Figure 4).

## 4. Discussion

Our results confirmed that tuning forks can be utilised to study the behavioural responses of orb web spiders. The behaviours demonstrated by *M. segmentata* are in accordance with the secondary defences described by Cloudsley-Thompson [10] and Tolbert [11] in that we observed both rebuff (attacking the stimuli) and flight (running away from the stimuli). The most common escape behaviour in our study was dropping from the web, which was also shown sometimes following attack behaviour. Dropping from the web on a safety thread to enable an easy return to the web after the danger has passed has been described as common in both araneid and tetragnathid spiders [10,38]. In the ethogram developed based on our study, we broadly categorised the behaviours as attack, escape, and no response, with the first group showing the largest number of distinct behaviours. Behaviours in this group also showed a combination of attacking and escaping, with some spiders moving towards the location of the tuning fork, and then escaping by dropping from the web. It should be stated that the spiders observed within this experiment never exhibited the common anti-predatory response of web shaking. Defined by Willey et al. [39] as “violent, large amplitude, movements of the web surface”, this behaviour has been observed in *Cyrtophora* [40] and *Mecynogea* [39]. Tolbert [11] called it web flexing, and observed the “Spider and web [swinging] back and forth parallel to the ground…” in *Argiope* and *Araneus* spp. This behaviour has been observed in the tetragnathid *Azilia vachoni* (Caporiacco, 1954) [38], but it is possible that the Meteine group of tetragnathids do not show this anti-predator behaviour.

The replication of results from three different methods, over two years, demonstrates the feasibility of obtaining reliable behavioural data using tuning forks, and the ease at which they can be used in the field confirm their suitability. The repetition in behavioural responses for the two tuning forks, regardless of the order the stimulus was presented, further demonstrates the reliability and effectiveness of the method. Directly stimulating spiders mechanically with a wire was, while still generating a clearly defined escape or attack behaviour, less reliable. Additionally, the behavioural responses to this stimulus appeared to be dependent on the order of its use; the change from attack to escape when it is the second and third stimuli could be due to the spider becoming sensitised to the prior stimuli. For *M. segmentata*, the 440 Hz tuning fork is the ideal frequency to simulate predators, as the behavioural responses to this frequency are principally attacking (90% of responses for both years combined). The 256 Hz tuning fork largely produces escape behaviours (58% of responses across the two years), although there was more variation with this tuning fork (36% showed attack behaviours). This could mean that a lower frequency is required to produce the ideal simulation of a predator as larger, and hence potentially more dangerous, insects tend to vibrate their wings between 100 Hz and 250 Hz [33]. Alternatively, this inter-individual variation could be attributed to behavioural syndromes, defined by Sih et al. [41] as “a suite of correlated behaviors reflecting between individual consistency in behavior across multiple (two or more) situations”. Behavioural syndromes can explain the tendency of some spiders to always attack or always escape from certain stimuli. For example, there are aggression syndromes, where across a range of situations, some individuals are more aggressive, whilst others are less aggressive [41]. The ease of using the tuning fork method in the field mean it could potentially be used to study behavioural syndromes in spiders in the wild. 

The size of the spider (here measured as total length) in our study did not significantly affect whether they responded to the stimulation with attack or escape behaviours, although there was a weak trend across the two tuning forks for the attacking spiders being slightly larger than those that escaped. Similarly, the size of the web (here measured as capture spiral area) did not differ between those spiders that attacked or escaped. However, again there was a weak trend for spiders showing attack behaviour in response to the tuning forks to have larger webs, although an opposite trend was found in response to the wire. Our findings are supported by a previous study relating female aggressiveness towards males and prey in the araneid spider *Larinioides sclopetarius* (Clerck, 1757), which found that aggressiveness was largely independent of female size [42].

Our findings further demonstrate how tuning forks can be used to collect quantitative data such as the spider’s reaction time to the presented stimuli. Reaction time has been a widely used measure for quantifying foraging behaviour [13,14], and it has also been used to estimate motivation or preparedness of the spider to repair webs under different wind loadings [16]. In our study, spiders showed the fastest reaction time to the wire, and the slowest responses to the 256 Hz tuning fork. However, interestingly the 256 Hz tuning fork elicited a number of spiders to react with escape behaviours before the tuning fork came into contact with the web, thereby producing a negative reaction time. This occurred across the 2020 and 2021 methodologies. This suggests that *Metellina* spiders are able to detect air-borne movements of predator species similar to reactions of airborne vibrations from prey demonstrated in araneid orb spiders [43]. This phenomenon can be explained by trichobothria, which are cuticular filiform hairs that respond to air movements and can be found on the legs and pedipalps of spiders [44,45]. 

The effectiveness of using tuning forks still needs to be investigated in more detail using more orb spider species and a wider range of frequencies in order to confirm our findings that show that orb web spiders tend to respond to high frequencies with prey capture behaviour, and to low frequencies with anti-predatory behaviours. Given that Nakata and Mori [29] found that both the small-to-medium-sized araneid *Cyclosa argenteoalba* (Bösenberg and Strand, 1906) (similar in size to *M. segmentata*) and the larger araneid *Eriophora sagana* (Tanikawa, 2000) show anti-predator responses to a 440 Hz tuning fork, it is clear that there are family-, environmental- or species-specific factors at play. However, even if the application of the methods in this study should turn out to be limited to Tetragnathid spiders in the Meteine group, the use of tuning forks could still be of potential use in studying the behaviour of Meteine cave spiders [46]. Studying the behaviour of cave animals in general is difficult due to the impediments caused by high sensitivity to disturbances, low population densities and the practical difficulties of observers spending a long time in caves [47]. So, developing easy and quick methods, such as using tuning forks, as demonstrated in this study, is essential. Anti-predatory behaviours are especially interesting to study in caves, as their low nutrient availability results in few species, and hence few predators, which again should reduce any anti-predatory responses [48]. Thus, comparing responses to the 256 Hz tuning fork at different distances into the cave and between spiders with different degrees of behavioural adaptations to the subterranean habitat, such as *Meta menardi* (Latreille, 1804), which modifies its orb web geometry [49], and *Metellina merianae* (Scopoli, 1763), which does not [50], would shed light on the interplay between evolution and phenotypic plasticity (flexibility in web-building behaviour in response to environmental factors) in shaping the behaviours of cave animals. 

## Figures and Tables

**Table 1:** Ethogram of anti-predatory and predatory behaviours in *Metellina segmentata*. The ethogram is split into three categories, attack, escape, and no response.

**Behavioural Response**		**Stimulus**	**No. of Responses**
Attack			
Turning towards the tuning fork	The spider, whilst staying on the central hub, turned to face the direction of the tuning fork, but did not move from the hub.	256 Hz and 440 Hz	2 (2020) 6 (2021)
Moving towards the tuning fork	The spider moved towards the location of the tuning fork, then stopped on the capture spiral, or returned to the central hub.	256 Hz and 440 Hz	23 (2020) 8 (2021)
Touching the tuning fork	The spider moved towards the tuning fork, and then touched it, but remained on the web.	440 Hz	1 (2020) 15 (2021)
Moving towards the tuning fork, then dropping from the web	The spider moved towards the tuning fork, but before touching it/getting close, the spider dropped from the web.	256 Hz and 440 Hz	17 (2020) 15 (2021)
Touching the tuning fork, then dropping from the web	The spider moved towards the tuning fork, after coming into contact with it, the spider dropped from the web.	440 Hz	6 (2020) 2 (2021)
Attacking	The spider moved its legs, to either grab or fight the wire, and remained on the web.	Wire	11 (2020) 12 (2021)
Grabbing	The spider held onto the wire, coming off the web.	Wire	5 (2020) 5 (2021)
Escape			
Dropping from the web	The spider dropped off the web; either to the floor, vegetation below, or in the air.	256 Hz, 440 Hz and wire	22 (2020) 27 (2021)
Moving/running away	The spider ran away, usually in the opposite direction to the tuning fork, moving off the web onto adjacent vegetation	256 Hz and wire	8 (2020) 8 (2021)
Jumping away	The spider jumped away after being touched by the wire, but remained on the web.	Wire	9 (2020) 10 (2021)
No response			
Flinching	The spider’s body moved slightly. It recoiled, but remained on the web.	Wire	6 (2020) 2 (2021)
No response	The spider did not respond to the stimuli in any way.	256 Hz, 440 Hz, wire	4 (2020) 7 (2021)

## Data Availability

Link to Figshare will be made available after manuscript acceptance (https://figshare.com/articles/dataset/Response_of_an_orb_spider_to_tuning_forks/19165460).

## Supplementary Materials

The following supporting information can be downloaded at: https://www.mdpi.com/article/10.3390/insects13040370/s1, Video S1: *Metellina segmentata* showing attack behaviour in response to a 256 Hz tuning fork. Video S2: *Metellina segmentata* showing escape behaviour in response to a 256 Hz tuning fork. Video S3: *Metellina segmentata* showing attack behaviour in response to a 440 Hz tuning fork. Video S4: *Metellina segmentata* showing attack behaviour in response to direct stimulation by a metal wire.
